# Evolution of hierarchical phase-contrast tomography on the European Synchrotron beamlines BM05 and BM18: a whole adult human brain imaging case study

**DOI:** 10.1107/S1600577526005503

**Published:** 2026-06-19

**Authors:** Hector Dejea, Joseph Brunet, Theresa Urban, Camille Berruyer, Alessandro Mirone, Christoph Jarnias, Alexandre Bellier, Claire Walsh, Peter D. Lee, Paul Tafforeau

**Affiliations:** a ESRF – The European Synchrotron, Grenoble, France; b Department of Mechanical Engineering University College London London United Kingdom; c Department of Anatomy (LADAF) Université Grenoble Alpes INSERM 1406 Grenoble France; Paul Scherrer Institut, Switzerland

**Keywords:** human brain, synchrotron, phase-contrast tomography, whole-organ imaging

## Abstract

Hierarchical phase-contrast tomography (HiP-CT) was recently developed to enable the *ex vivo* imaging of human organs at multiple scales from whole organ down to near-cellular resolution in localized regions. Using whole adult human brain imaging as a case study, this article shows the evolution and optimization of this technique from its initial development at the BM05 beamline to its transition and current status at BM18. Thanks to the higher spatial coherence, larger beam size, higher energies and larger propagation distances available at BM18 and due to the European Synchrotron’s Extremely Brilliant Source upgrade (ESRF-EBS), this transition resulted in significantly improved data quality, resolution, sensitivity and speed. More recently, the implementation of a new generation of larger sCMOS cameras, helical scanning (including dedicated reconstruction algorithm developments), binning at the chip and projections levels, and the design of high-efficiency optics allowed us to progressively improve the trade-off between dose and image quality, and acquisition time. These advances enable whole-organ imaging at voxel sizes ranging from ∼42 µm to ∼15 µm, with acquisition times reduced from ∼18 h to ∼3–6 h, depending on configuration. These acquisition schemes present the current status of full-organ imaging using HiP-CT and represent the constant efforts for improvement of the technique towards the investigation of human organs in health, disease and ageing.

## Abstract 2

The evolution of hierarchical phase-contrast tomography (HiP-CT) from its origins at the BM05 beamline to its transition to BM18 (ESRF-EBS) is shown. The novel hardware and scanning approach developments resulted in significantly improved data quality, resolution, sensitivity and speed. The case of whole adult brain imaging is presented to demonstrate the current possibilities of full-organ imaging with local micrometre resolution using HiP-CT.

## 1. Introduction

With the wide availability of third-generation synchrotron facilities during the last decades, propagation-based X-ray phase-contrast tomography has become a staple methodology for non-destructive, 3D, high-resolution studies in a broad variety of fields, such as biomedicine, palaeontology, earth and material sciences, among others.

Specially for biomedical applications, X-ray phase-contrast imaging has been able to overcome the limitation of resolving low absorption contrast in soft tissues by exploiting the differences in refractive index, which are orders of magnitude more sensitive in the typical energy range used for X-ray imaging (Lewis, 2004 ▸). Propagation-based phase-contrast imaging has been applied to study a multitude of organs down to the cellular level: lungs (Lovric *et al.*, 2013 ▸; Norvik *et al.*, 2020 ▸; Borisova *et al.*, 2021 ▸; Fardin *et al.*, 2021 ▸), heart (Mirea *et al.*, 2015 ▸; Gonzalez-Tendero *et al.*, 2017 ▸; Dejea *et al.*, 2019 ▸; Wang *et al.*, 2019 ▸; Reichardt *et al.*, 2020 ▸; Planinc *et al.*, 2021 ▸), brain (Beltran *et al.*, 2011 ▸; Massimi *et al.*, 2019 ▸; Longo *et al.*, 2021 ▸; Bosch *et al.*, 2022 ▸; Barbone *et al.*, 2020 ▸), musculoskeletal tissues (Cooper *et al.*, 2011 ▸; Disney *et al.*, 2019 ▸; Pierantoni *et al.*, 2021 ▸; Madi *et al.*, 2020 ▸; Dejea *et al.*, 2024 ▸; Horng *et al.*, 2014 ▸), pancreas (Tapfer *et al.*, 2013 ▸; Frohn *et al.*, 2020 ▸; Pinkert-Leetsch *et al.*, 2023 ▸), and many others. However, these studies have been limited to small animal (usually rodent) organs or tissue biopsies of human or large animals due to the technical specifications of available beamlines, primarily small beam sizes (generally < 50 mm × 10 mm) and limited propagation distances, without the level of coherence required to avoid image blurring at long propagation distances. Other facilities have also developed large-field imaging capabilities for biological samples, such as BL20B2 at SPring-8 (Saito *et al.*, 2020 ▸), which provides wide-beam X-ray imaging and phase-contrast tomography. However, despite the large beam size, such beamlines are generally applied to samples of the order of a few centimetres or localized regions of larger specimens rather than full intact adult human organs. Similarly, alternative phase-contrast techniques which are less demanding in terms of coherence and propagation, such as grating interferometry, analyser-based imaging, speckle tracking and edge illumination (Keyriläinen *et al.*, 2005 ▸; Beltran *et al.*, 2011 ▸; Bravin *et al.*, 2013 ▸; Diemoz *et al.*, 2016 ▸; Kaneko *et al.*, 2017 ▸; Longo *et al.*, 2024 ▸; Riedel *et al.*, 2021 ▸). However, these methods are dose-inefficient compared with propagation-based techniques, require more cumbersome setups and have yet to image intact adult organs at micrometre resolution. More fundamentally, operating at high energies while maintaining sufficient flux remains a key limitation for many propagation-based imaging beamlines.

With the recent, current and near-future upgrade of light sources around the world into fourth-generation synchrotrons, such as the Extremely Brilliant Source upgrade at the European Synchrotron (ESRF-EBS), highly coherent [100× higher than the former ESRF (Pacchioni, 2019 ▸)] high-flux beams have enabled high-energy full-field tomography beamlines to achieve propagation distances reaching in some cases the near-field limit for single-distance propagation without being limited by the coherence, so that the capability to resolve small density differences has been dramatically increased. At the ESRF-EBS this has been especially significant for previous bending-magnet beamlines, which have been substituted by short two- or three-pole wigglers and can now take full advantage of the upgrade benefits.

Taking advantage of these developments, hierarchical phase-contrast tomography (HiP-CT) (Walsh *et al.*, 2021 ▸) was developed to enable the imaging of adult human organs at multiple scales from whole organ down to near-cellular level. HiP-CT has already been successfully applied to the study of several adult human organs in health and disease, such as lung (Ackermann, Kamp *et al.*, 2022 ▸; Ackermann, Tafforeau *et al.*, 2022 ▸; Verleden *et al.*, 2024 ▸), heart (Brunet *et al.*, 2024 ▸), placenta (Reichmann *et al.*, 2024 ▸) or temporal bone (Schaeper *et al.*, 2025 ▸).

HiP-CT was initially developed at the ‘X-ray Imaging and Optics Test’ beamline BM05 (ESRF-EBS), which is limited to a maximum propagation distance of 3.5 m and a beam size of 50 mm × 4 mm at the sample position (52 m). However, the recent beamline for ‘Hierarchical Tomography’ BM18 (ESRF-EBS) (Lang *et al.*, 2023 ▸; Cianciosi *et al.*, 2023 ▸; Vijayakumar *et al.*, 2024 ▸) takes full advantage of the enhanced EBS brilliance properties by allowing up to 36 m of propagation in a 220 m-long beamline, with a filtered polychromatic beam (38–300 keV) of 300 mm × 17 mm at the sample position (178 m). See Table 1 ▸ for comparative technical specifications of both beamlines.

In this article, we report on developments enabling the evolution of HiP-CT data quality, resolution and sensitivity. These have been enabled by the transition from BM05 to BM18, as well as the incorporation of new hardware, alternative acquisition approaches and software developments. This is demonstrated by using the case study of whole adult human brain imaging.

## 2. Material and methods

### 2.1. Organ and donor description

A whole control human brain was obtained from a body donation to the Department of Anatomy of Grenoble Hospital (Laboratoire d’anatomie des alpes françaises, LADAF). The donor LADAF-2021-17 was a 63-year-old, 178 cm, 60 kg male with pancreatic cancer. During autopsy, stomach hypertrophy and aspiration of gastric contents were noted.

Body donation is based on a voluntary act of free consent by the donors *antemortem*. The dissection was performed respecting the memory of the deceased and following current French legislation for body donation. The post-mortem study was conducted according to the Quality Appraisal for Cadaveric Studies scale recommendations (Wilke *et al.*, 2015 ▸).

### 2.2. Organ preparation

After death, the donor body was embalmed by injecting formalin-based embalming solution into the right carotid artery. The body was then stored at 3.6°C for a few days. The dissection, including the extraction of the brain, was performed at the LADAF. The brain was then post-fixed in 4% neutral buffered formalin at 3.6°C for at least 4 days. Following the sample preparation protocol described by Brunet *et al.* (2023 ▸), the brain underwent partial dehydration using increasing ethanol concentration baths until 70% ethanol was reached (4 days per bath at 50%, 60%, 70% and 70% ethanol concentration). The brain was stabilized in equilibrated crushed agar–ethanol gel with the frontal cortex facing downwards and degassed using thermal cycling (Brunet *et al.*, 2023 ▸). The container was finally sealed and stored at room temperature until scanning (Fig. S1 of the supporting information). A minor shrinkage was observed over extended storage periods, likely related to ethanol conservation rather than the imaging process.

### 2.3. Imaging protocols

To assess the improvement in HiP-CT data quality and compare each beamline, the brain was initially imaged at BM05 using a 25.25 µm voxel size configuration. It was later imaged at BM18 using two configurations at 42.4 µm and 23.42 µm voxel size. Following the technical evolution of the BM18 beamline described below, the same brain was imaged again with consecutive developments at 19.28 µm/voxel, 20.17 µm/voxel and 14.29 µm/voxel. For clarity, these acquisition setups are hereafter referred to as Configurations 1–6. Configuration 1 corresponds to BM05 (25.25 µm), while Configurations 2–6 correspond to the successive BM18 setups. Due to the large source-to-sample distances at both BM05 (∼52 m) and BM18 (∼178 m), the beam divergence remains very low (microradian range), resulting in negligible geometric effects across the sample. Therefore, the quasi-parallel beam approximation is valid for both beamlines, even for large samples such as whole human organs. The spatial coherence length, at 100 keV, was approximately ∼5 µm for BM05 and ∼18 µm for BM18. The propagation distance was selected as a trade-off between phase-contrast sensitivity and photon flux, considering beam energy, voxel size and experimental constraints. While the near-field regime provides a useful guideline, the selected distances were adapted in practice to optimize image quality and acquisition efficiency. The technical specifications of the beamlines are summarized in Table 1 ▸, a detailed list of all setups and acquisition parameters can be found in Table S1 of the supporting information and a timeline of development can be found in Fig. S2. The detected spectra, corresponding to each configuration, are provided in Fig. S3. Dose rates and integrated absorbed doses were estimated using a beamline simulation calibrated with experimental measurements, as detailed in the supporting information.

#### 2.3.1. BM05 – initial development (Configuration 1)

The brain was initially imaged using HiP-CT at the BM05 beamline at the European Synchrotron (ESRF-EBS, Grenoble, France). This dataset can be found at https://doi.org/10.15151/ESRF-DC-1773964937.

The scanning protocol of Configuration 1 consisted of using a quasi-parallel polychromatic beam attenuated by 0.23 mm of copper and 0.68 mm of molybdenum. Propagation-based full-field tomography of the whole brain was performed in quarter-acquisition mode at a voxel size of 25.25 µm with a propagation distance of 3.5 m, 9900 projections per scan and 36 ms exposure time per projection (accumulation of 6 times 6 ms). The quarter-acquisition protocol consists of a combination of a standard half-acquisition and an additional annular scan to extend the field of view (Walsh *et al.*, 2021 ▸). In this approach, projections from the two acquisitions are concatenated horizontally prior to reconstruction, with normalization applied in the overlapping regions to ensure consistent grey levels. The reconstruction then follows the same pipeline as a conventional acquisition. This strategy enables imaging of samples as large as four times the detector field of view while avoiding the complexity of mosaic scanning. A total of 75 pairs of scans were required to cover the whole brain vertically. X-rays were converted to visible light by a LuAG:Ce 1000 µm scintillator (Crytur, Czech Republic), demagnified by a Dzoom optic (×0.25) and detected by a PCO Edge 4.2 CLHS detector (2048 × 2048 pixels of 6.5 µm, PCO imaging, Germany). This configuration resulted in an average detected energy of 97 keV after absorption by the sample. The total acquisition time was 18.3 h.

#### 2.3.2. BM18 – improvement of data quality due to long-propagation phase contrast (Configuration 2 and 3)

The brain was also imaged using HiP-CT in five different configurations at the BM18 beamline at the European Synchrotron (ESRF-EBS, Grenoble, France) from 2021 to 2025 as the technique evolved.

The scanning protocols included the use of a filtered quasi-parallel polychromatic beam. Configuration 2 was performed overnight on the first day of tomography on the new BM18 beamline. Propagation-based full-field tomography of the whole brain was achieved with the use of a 1.3 mm molybdenum attenuator, in half-acquisition mode at a voxel size of 42.4 µm with a propagation distance of 38 m, 6000 projections per scan and 105 ms exposure time per projection (accumulation of 7 times 15 ms). A total of 24 vertical scans were required to cover the full volume of the brain. X-rays were converted to visible light by a LuAG:Ce 2000 µm scintillator (Crytur, Czech Republic), demagnified by the LAFIP2 optic (tandem combining a 400 mm custom objective with a Canon 50 mm one, leading to a ×0.125 magnification) and detected by a PCO Edge 4.2 CLHS detector (PCO imaging, Germany). This configuration resulted in an average detected energy of 122 keV after absorption by the sample. The total acquisition time was 5.7 h. This dataset can be found at https://doi.org/10.15151/ESRF-DC-2313098569.

Configuration 3 was performed in February 2022 right after the implementation of the Dzoom optic on BM18 (similar to that used on BM05). The organ jar was immersed in 70% ethanol (5 mm thickness) in a surrounding rig tube to guarantee a cylindrical shape and to allow the complete jar to be included in the field of view without artefacts coming from the interface with air. Propagation-based full-field tomography was performed with 0.61 mm of molybdenum, in quarter-acquisition mode at a voxel size of 23.42 µm with a propagation distance of 31 m, 9990 projections per scan and 45 ms exposure time per projection (accumulation of 3 times 15 ms). A total of 33 vertical scans were required to cover the whole brain. X-rays were converted to visible light by a LuAG:Ce 2000 µm scintillator (Crytur, Czech Republic), demagnified by the Dzoom optic and detected by a PCO Edge 4.2 CLHS detector (PCO imaging, Germany). This configuration resulted in an average detected energy of 110 keV after absorption by the sample. The total acquisition time was 8.9 h. Regarding the detector configuration, with the exception of the thickness of the scintillator which was twice higher on BM18 than on BM05, the two detector systems can be considered identical. This dataset can be found at https://doi.org/10.15151/ESRF-DC-1773964905.

#### 2.3.3. BM18 – improving dose efficiency (Configuration 4)

Configuration 4 focuses on improving dose efficiency for whole-organ imaging. The key element was the use of binning at the detector level for an increased detection efficiency (4 pixels in the place of 1), thus reducing the dose required for imaging. Detector-level binning is performed on-chip during acquisition, improving the signal-to-noise ratio and enabling dose reduction. Moreover, the addition of large-field detectors (Iris 15 Scientific CMOS, Teledyne Photometrics, USA with 5056 × 2960 pixels of 4.25 µm) still allowed coverage of the complete brain in quarter-acquisition mode. Across the different configurations, two sCMOS detector systems were used (PCO Edge 4.2 CLHS and Iris 15). A detailed comparison of their key specifications and performance is provided in Table S2.

The organ jar was also immersed in 70% ethanol (5 mm thickness). Propagation-based full-field tomography was performed with 10 mm of sapphire, 0.3 mm of silver and 30 mm of glassy carbon, in quarter-acquisition mode at a voxel size of 19.28 µm (9.64 µm in bin 2) and with a propagation distance of 20 m, 12000 projections per scan and 36 ms exposure time per projection (accumulation of 3 times 12 ms). A total of 24 vertical scans were required to cover the whole brain. X-rays were converted to visible light by a GAGG 1000 µm scintillator with reflective layer (Crytur, Czech Republic), demagnified by a Dzoom optic and detected by an Iris 15 Scientific CMOS detector (Teledyne Photometrics, USA). The scintillator thickness and type were selected to maximize light output while preserving spatial resolution, considering the numerical aperture of the optical system and detector pixel size. In later configurations, thinner scintillators with a reflective coating were used to improve light collection efficiency while maintaining spatial resolution. This configuration resulted in an average detected energy of 103 keV after absorption by the sample. The total acquisition time was 6.8 h. This dataset can be found at https://doi.esrf.fr/10.15151/ESRF-DC-2313101083.

#### 2.3.4. BM18 – improving acquisition speed (Configuration 5)

In Configuration 5, the key additions were the use of helical scanning, which removes overheads in individual scans and improves concatenation artefacts, as well as the use of bin mean, corresponding to binning performed at the projection level using averaging rather than on-chip pixel summation. In this context, projection-level binning is applied after acquisition by averaging neighbouring pixels during processing. Compared with on-chip binning, this approach does not provide intrinsic dose efficiency gains since the dynamic range of each individual pixel is used. Instead, the overall dose reduction (approximately a factor of 2) arises from the reduced number of projections.

The organ jar was also immersed in 70% ethanol (5 mm thickness). Propagation-based full-field tomography was performed with 10 mm of sapphire, 0.4 mm of silver and 35 mm of glassy carbon, in quarter-acquisition mode at a voxel size of 20.17 µm (10.08 µm in bin mean 2) and with a propagation distance of 11 m, 12000 projections per scan and 12 ms exposure time per projection. A total of 35 vertical turns were required to cover the whole brain. X-rays were converted to visible light by a GAGG 1000 µm scintillator with reflective layer (Crytur, Czech Republic), demagnified by a Dzoom optic and detected by an Iris 15 Scientific CMOS detector (Teledyne Photometrics, USA). This configuration resulted in an average detected energy of 113 keV after absorption by the sample. The total acquisition time was 3.2 h. This dataset can be found at https://doi.org/10.15151/ESRF-DC-2313101075.

#### 2.3.5. BM18 – best configuration with fixed ×0.5 magnification optics (Configuration 6)

Configuration 6 takes advantage of the latest addition of optics at BM18, a fixed-magnification tandem optic at ×0.5 (Mamiya 200 mm f/d 2.8 / Otus 100 mm f/s 1.4). The term ‘fixed magnification’ refers to an optical system with a constant magnification factor, in contrast to zoom optics, resulting in improved light collection efficiency and image quality. This configuration improves significantly the optical efficiency compared with the previously used Dzoom. Based on optical calculations, the gain is approximately a factor of 22 for identical scintillator configurations, while experimental measurements indicate an overall signal gain of about 8 to 10 when combined with thinner scintillators (250um GAGG+ with reflective layer), depending on beam energy. No significant distortion or vignetting artefacts were observed with this optical configuration across the field of view.

The organ jar was also immersed in 70% ethanol (5 mm thickness). Propagation-based full-field tomography was performed with 5 mm of sapphire and 0.5 mm of silver, in quarter-acquisition mode at a voxel size of 14.79 µm (7.39 µm in bin mean 2) and with a propagation distance of 20 m, 15000 projections per scan and 18 ms exposure time per projection. A total of 39 helical turns were required to cover the whole brain. X-rays were converted to visible light by a GAGG 250 µm scintillator with reflective layer (Crytur, Czech Republic), demagnified by a fixed ×0.5 magnification optic and detected by an Iris 15 Scientific CMOS detector (Teledyne Photometrics, USA). This configuration resulted in an average detected energy of 102 keV after absorption by the sample. The total acquisition time was 6.5 h. This dataset can be found at https://doi.org/10.15151/ESRF-DC-2313101091.

### 2.4. Tomographic reconstruction and data visualization

Flat-field correction was performed using reference scans acquired before and after sample imaging in a container filled with agar and 70% ethanol under identical acquisition conditions. The flat-field image was computed as the average of all projections from these reference scans.

Configurations 1–4 were reconstructed following the pipeline described in detail by Xian *et al.* (2022 ▸). Briefly, the reconstruction consisted of a filtered back-projection algorithm in combination with the single-distance phase retrieval algorithm by Paganin *et al.* (2002 ▸) and 2D unsharp filtering using *PyHST2* (Mirone *et al.*, 2014 ▸). For Configurations 1 and 2, the reconstructed volumes were vertically concatenated to obtain the full brain volume. For all subsequent acquisitions, the vertical concatenation was instead performed at the projection level by stitching projections at each angle prior to reconstruction, forming a single extended dataset. This approach increases computational demands but avoids artefacts associated with post-reconstruction stitching. Ring artefact correction was then performed on all reconstructed datasets using an algorithm based on the work of Lyckegaard *et al.* (2011 ▸).

The two final helical scans (Configurations 5 and 6) were reconstructed using the ESRF tomography processing software *Nabu* together with the *night_rail* framework (Mirone, 2025 ▸), which enables reconstruction of continuously acquired helical data. Reconstruction was performed using a filtered back-projection algorithm in combination with the single-distance phase retrieval method by Paganin *et al.* (2002 ▸) and 2D unsharp filtering.

Finally, the reconstructed volumes were cropped and converted to JPEG2000 format to reduce data size and storage requirements, while preserving image quality for downstream analysis.

The presented data have been registered and visualized using *VGSTUDIO MAX 2024.1*. All the reconstructions are available free to access at the Human Organ Atlas portal (https://human-organ-atlas.esrf.fr) (Walsh *et al.*, 2025 ▸).

## 3. Results

**Figure 1:**
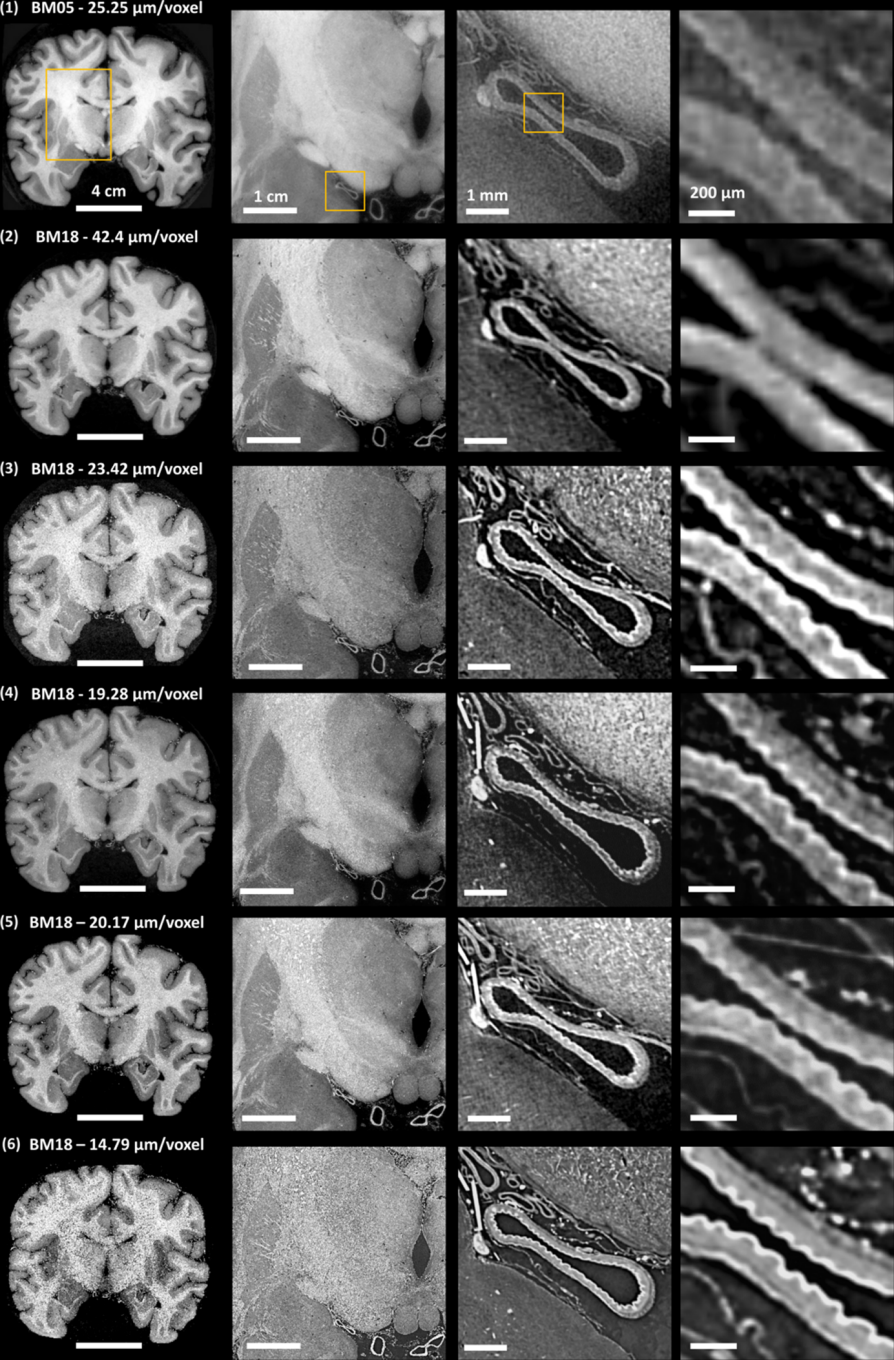
Comparison between BM05 and BM18 acquisitions using an illustrative coronal slice at the same location of the brain. From left to right column: complete slice, first, second and third digital zoom levels in one of the main arteries. Zoom locations are depicted in yellow in the top row and remain equal across acquisition types. Note that acquisition parameters differ between configurations (voxel size, propagation distance, detector setup), reflecting the progressive optimization of the technique. Contrast levels were adjusted independently for each dataset to optimize visualization of anatomical structures.

Figs. 1 ▸, 2 ▸ and 3 ▸ illustrate the improvement in HiP-CT data quality by comparing Configuration 1 (BM05, 25.25 µm/voxel) with Configurations 2 to 6 at BM18, all acquired on the same whole adult human brain (LADAF-2021–17), at 42.4 µm/voxel, 23.42 µm/voxel, 19.28 µm/voxel, 20.17 µm/voxel and 14.79 µm/voxel, respectively, each of these measurements representing a specific improvement of the technique. The figures present data in the coronal, sagittal and transversal planes, respectively.

Configuration 1 (BM05, 25.25 µm/voxel) shows good contrast between white and grey matter, but is unable to resolve structures within the tissue other than the ventricles and very large vessels. This is due to the very short propagation distance, which does not allow small structures with weak phase shifts to generate sufficient phase fringes, thus losing sensitivity. In Configuration 2 (BM18, 42.4 µm/voxel), the improvement in data quality is already very clear, with preserved contrast and enhanced visualization of internal structures and tissue boundaries. Using a comparable voxel size, Configuration 3 (BM18, 23.42 µm/voxel) shows a marked improvement in sensitivity, enabling even different arterial sub-layers (intima, media, adventitia) in large vessels to be resolved (Fig. 1 ▸).

The incorporation of large chip detectors at BM18 (Iris15, 5056 × 2960 pixels, 4.25 µm/pixel) allowed coverage of much larger field of views at smaller voxel size. Initially in combination with chip binning, and later with helical scanning and binning at the projection level, the dose deposited could be reduced while maintaining imaged quality and resolving very similar structures (Configurations 4 and 5, Figs. 1 ▸–3 ▸ ▸).

Finally, Configuration 6, using a fixed-magnification ×0.5 optic, clearly shows a great improvement in image quality while again reducing the dose required. This can be clearly noted by the sharpness of structures in vasculature, grey–white matter and cerebellum (Figs. 1 ▸–3 ▸ ▸).

These qualitative observations are supported by quantitative estimates of effective spatial resolution and image quality, as summarized in Table S3. The methodology is detailed in the supporting information.

## 4. Discussion

This article presents the progressive improvement of HiP-CT data quality by transitioning from its original development on beamline BM05 to BM18 (ESRF-EBS), which is purposely designed for multi-resolution phase-contrast imaging. It also shows the technique’s optimization to lower dose and scanning times thanks to the incorporation of new hardware and software tools, as well as alternative acquisition schemes (Fig. S2).

**Figure 4:**
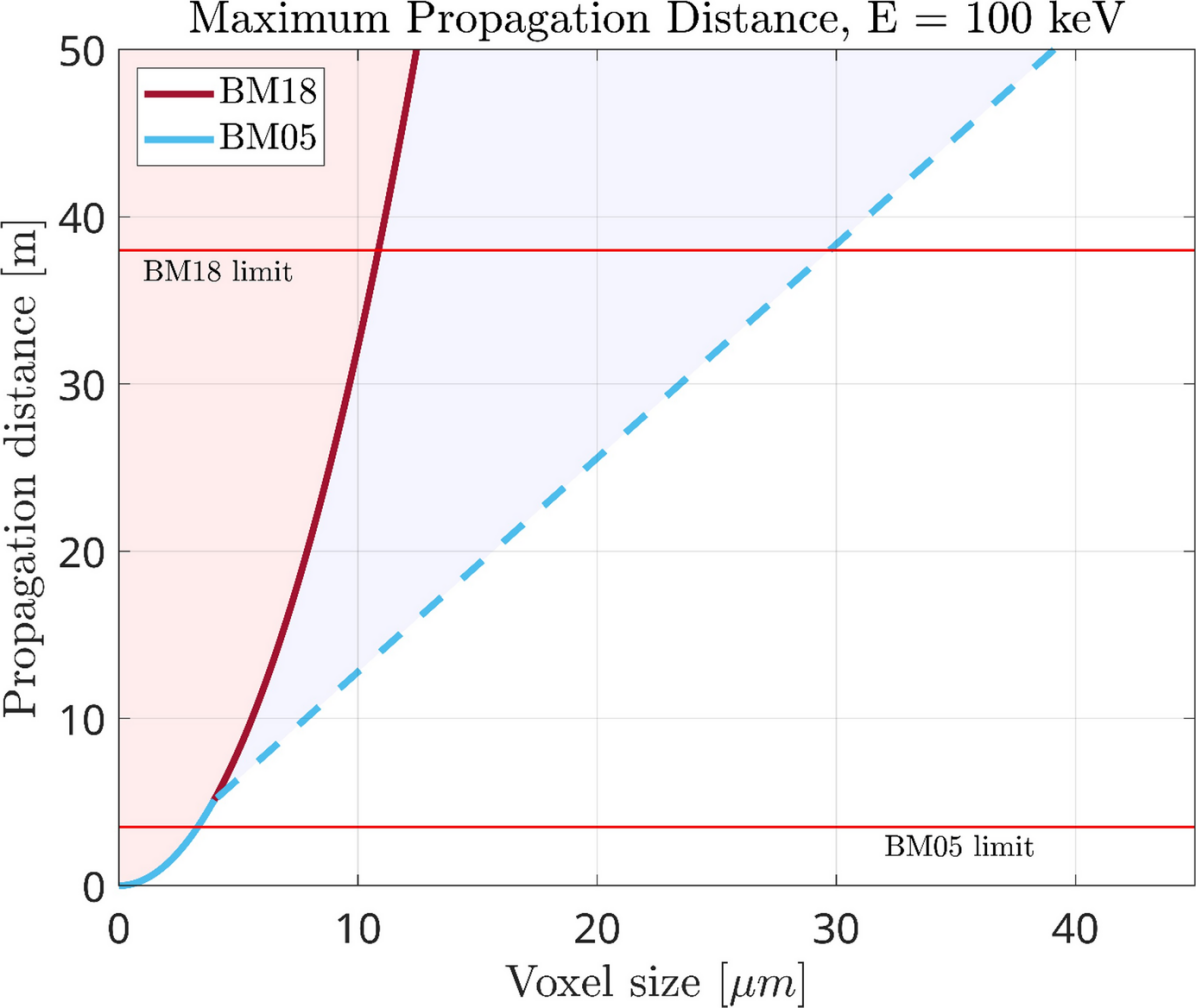
Maximum propagation distance achievable at beamlines BM18 and BM05 for an energy of 100 keV, as constrained by either the near-field limit or geometrical blur (independent of energy). The horizontal red lines indicate the maximum propagation distance achievable by hutch size. The dashed curve indicates the region at which geometrical blur takes over the near-field limit, which does not happen in BM18 until much further propagation distances than plotted. Forbidden zones are shadowed with the corresponding beamline colour.

The technical specifications of BM05, a relatively short beamline in comparison with BM18 (52 versus 178 m source–sample distance) and limited propagation distance (maximum 3.5 versus 36 m), make it ideal for phase-contrast imaging below ∼4 µm. Assuming an energy of 100 keV, the near-field limit is constrained by the hutch size at 3.3 µm/voxel, and if the hutch was larger, geometrical blurring will already be dominating at a propagation distance of 5 m for voxel sizes larger than 4 µm (Fig. 4 ▸). This is not the case for BM18, which allows propagation distances very close to the near-field limit without reaching geometrical blurring, so that the limitation becomes the size of the experimental hutch rather than the horizontal angular source size (Fig. 4 ▸). Assuming an energy of 100 keV, a propagation distance of 59.4 m would be required for geometrical blurring to dominate over the near-field limit for voxel sizes above 13.5 µm, a regime that remains inaccessible within the current hutch dimensions. See the supporting information and Fig. S4 for more details on near-field limit and geometrical blur calculations.

As a consequence, the specifications of BM18 make it the best currently existing beamline in the world to perform HiP-CT on samples as large as or even larger than adult human organs, which is clearly visible by the comparison in Figs. 1 ▸, 2 ▸ and 3 ▸. The larger propagation distance at such a level of coherence is able to take full advantage of the phase-contrast effect and amplify smaller structures, so that the overall data quality and sensitivity are improved for similar acquisition in both beamlines.

While BM18 is proven here to show a remarkable superiority in whole-organ imaging, BM05 remains a very powerful instrument for sub-micrometre voxel size imaging thanks to its higher power density (at <140 keV) and should always be considered for micrometre-resolution studies, as long as the propagation distance and smaller beam size do not become limitations.

**Figure 5:**
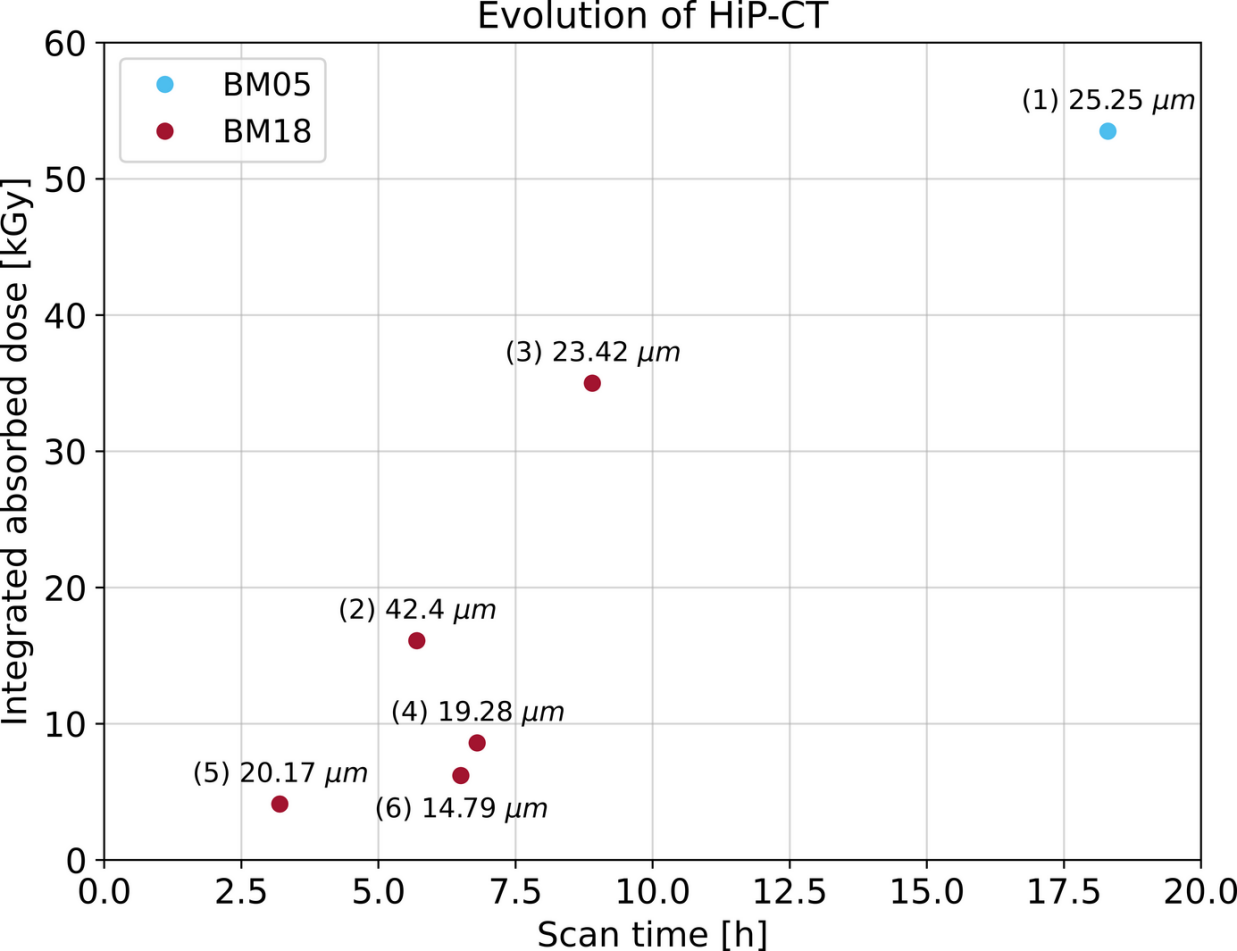
Relationship between integrated absorbed dose and total scanning time for the presented setups at beamlines BM18 and BM05. Each data point is marked by the setup number in order of acquisition and the corresponding voxel size.

After the transition from BM05 to BM18, the development of the technique has focused on the optimization of image quality with minimum dose deposition and scanning times. Large chip detectors with smaller pixel size (Iris15) were incorporated with the goal of improving resolution and data quality while reducing the dose and data size, which could be achieved by working in binning at the chip level. This allowed image quality to be maintained while reducing the dose by ∼75% (Table S1 and Fig. 5 ▸). However, this acquisition protocol still required the use of accumulation to reduce the noise level, which could be avoided by the use of binning by average at the projection level, instead of at the chip level (bin mean). This means that with a single shot, it is possible to achieve a dynamic level corresponding to an accumulation of 4 (coming from the 4 averaged pixels) without saturating the 32 bits dynamic scale of the pictures. Additionally, in the meantime, the possibility to perform helical scanning was developed at BM18, thus allowing reduction of scanning times by avoiding overheads in each stacked scan and obtaining an equivalent of accumulation 2 by doing a 50% overlap between helical turns. These advancements resulted in an approximately 50% reduction in both total scanning time and integrated absorbed dose, as summarized in Fig. 5 ▸.

The final step corresponds to a major improvement in image quality and sensitivity enabled by the introduction of a fixed magnification ×0.5. This optic is approximately 32 times more efficient than the previously used DZoom in terms of photon collection, enabling substantially higher sensitivity and improved image quality at smaller voxel sizes. In practice, this gain is primarily invested in improving spatial resolution and contrast, and acquisition speed rather than reducing dose, allowing a reduction of the voxel size from approximately 20 µm to 15 µm while maintaining comparable dose levels. This improvement arises primarily from the much larger numerical aperture of the fixed ×0.5 optic compared with the DZoom system (NA ≃ 0.2 versus 0.042), leading to a strongly enhanced light collection efficiency. Additional gains result from improved optical transmission and the use of more efficient scintillators (GAGG+ instead of LuAG:Ce). The higher numerical aperture also improves the diffraction-limited resolution, implying the use of thinner scintillators which brings higher sensitivity and image sharpness. As a result, this configuration becomes a game changer for the scanning of organ overviews with maximum efficiency.

This article has shown the developments, current status and possibilities of whole-organ imaging with HiP-CT after its successful transition from BM05 to BM18, using the same adult human brain as a case study. This evolution in configurations presents a reduction in integrated dose values (Fig. 5 ▸), which is very beneficial to reduce the risk of bubble formation (Xian *et al.*, 2024 ▸) and radiation-induced effects that could impact downstream ‘omics’ analyses, consistent with previous studies demonstrating preservation of histological and molecular integrity following X-PCI imaging (Li *et al.*, 2023 ▸). To our knowledge, no other imaging technique is able to provide whole adult human organ data in 3D, non-destructively, and at such spatial resolution and speed. That said, cumulative data size (∼1 TB per organ) and processing or analysis, which is currently highly manual and therefore time-consuming, remain a bottleneck. To tackle this, more efforts need to be put into the development of artificial intelligence methods and tools that can speed up the process while providing reliable quantification (Greenspan *et al.*, 2016 ▸; Hesamian *et al.*, 2019 ▸; Yagis *et al.*, 2024 ▸).

Currently, HiP-CT is fully operational at both BM05 and BM18 beamlines of the ESRF-EBS. In the near future, on-going hardware developments, such as the recently commissioned big sample stage (maximum 300 kg sample, 2.3 m vertical translation, 1.2 m sample diameter) in combination with existing optics that can take the full 30 cm of the beam, will allow imaging of larger multi-organ complexes. In addition, the recent installation of a PCO Edge 10 bi CLHS detector (4416 × 2368 pixels of 4.6 µm, PCO imaging, Germany) coupled to the fixed-magnification ×0.5 optics enables data acquisition with comparable image quality while reducing scan times by approximately a factor of 3 for the same dose, significantly improving throughput for large samples. In parallel, the later addition of the next-generation ‘Large Area Detector’ (LAD) from Fraunhofer IIS, with a lateral size of 16000 pixels coming from the combination of nine sCMOS chips, would enable the possibility to image full *ex-vivo* human bodies at 20 µm/voxel. In addition, the upgrades of other light sources to fourth generation, such as APS, SLS and DLS, or the construction of new beamlines such as the Biomedical Imaging Long Beamline project in South Korea, open the possibility of translating this technique for large organs to other facilities.

## 5. Related literature

The following reference, not cited in the main body of the paper, has been cited in the supporting information: Salditt *et al.* (2017 ▸).

## Supplementary Material

Supplementary Table S1. Summary of all HiP-CT datasets acquired for the human brain across BM05 and BM18 beamlines. The table includes acquisition parameters (voxel size, propagation distance, exposure), scanning configurations, and corresponding dataset links (DOIs) for each experiment. DOI: 10.1107/S1600577526005503/gy5090sup1.xlsx

Supporting Sections S1 to S3, inlcuding Tables S2 and S3, and Figures S1 to S4. DOI: 10.1107/S1600577526005503/gy5090sup2.pdf

## Figures and Tables

**Figure 2:**
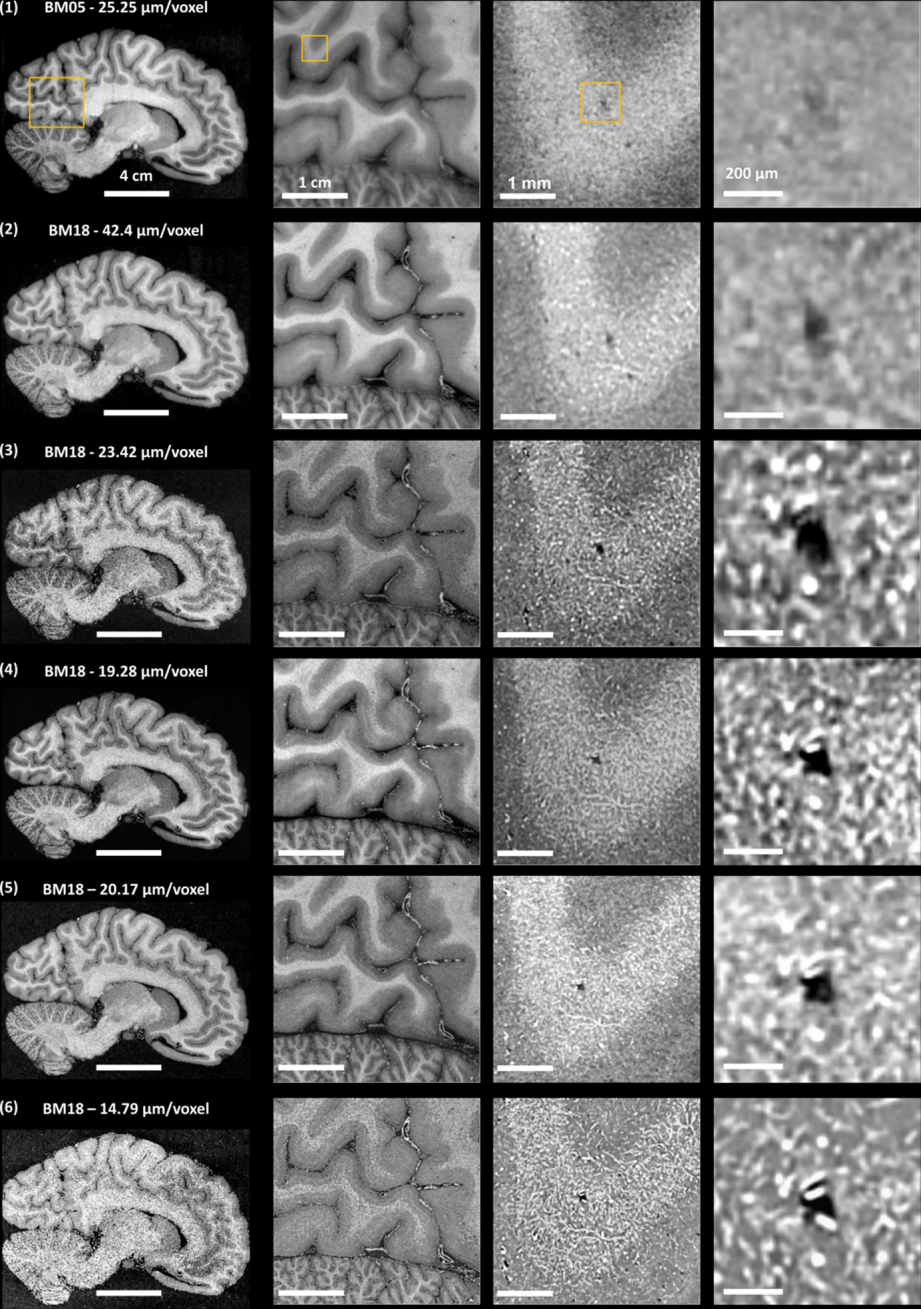
Comparison between BM05 and BM18 acquisitions using an illustrative sagittal slice at the same location of the brain. From left to right column: complete slice, first, second and third digital zoom level in the cortex containing both grey and white matter. Zoom locations are depicted in yellow in the top row and remain equal across acquisition types. Note that acquisition parameters differ between configurations (voxel size, propagation distance, detector setup), reflecting the progressive optimization of the technique. Contrast levels were adjusted independently for each dataset to optimize visualization of anatomical structures.

**Figure 3:**
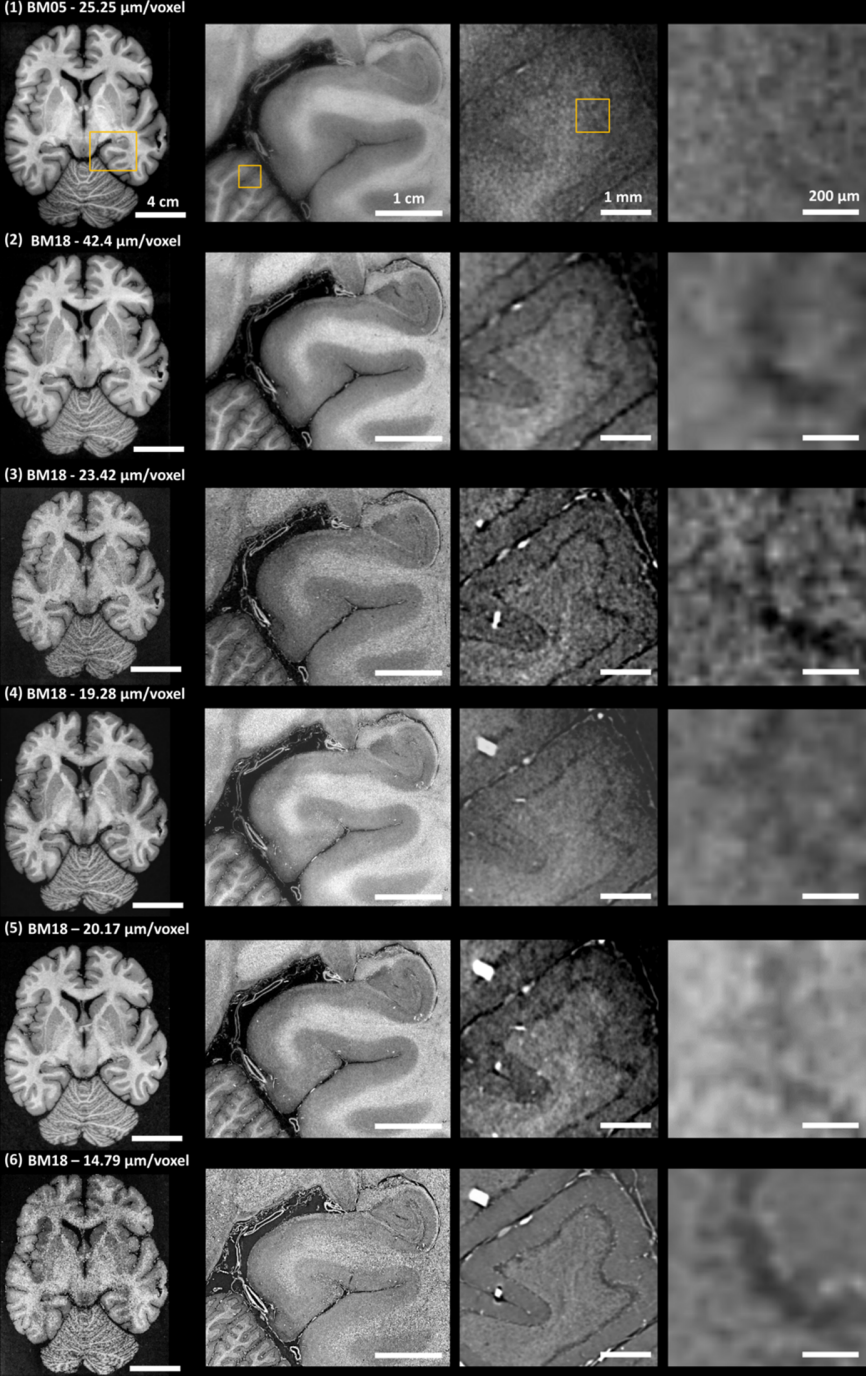
Comparison between BM05 and BM18 acquisitions using an illustrative transversal slice at the same location of the brain. From left to right column: complete slice, first, second and third digital zoom level of the cerebellum. Zoom locations are depicted in yellow in the top row and remain equal across acquisition types. Note that acquisition parameters differ between configurations (voxel size, propagation distance, detector setup), reflecting the progressive optimization of the technique. Contrast levels were adjusted independently for each dataset to optimize visualization of anatomical structures.

**Table 1:** Technical specifications of BM05 and BM18 beamlines (ESRF-EBS)

	BM05	BM18
Insertion device	Dipole wiggler (0.85 T peak field)	Tripole wiggler (1.56 T peak field, 1.08 T on lateral poles)
Energy range usable in polychromatic beam for tomography	25–150 keV	38–300 keV
Source–sample distance	52 m	178 m†
Sample–detector distance	0–3.5 m	0–32 m†
Beam size at sample position (h × v)	72 mm × 4 mm	300 mm × 17 mm
Horizontal angular source size	1.17 µrad	0.34 µrad
Voxel size range	0.35–26 µm	0.7–120 µm

† The source-to-sample distance and maximum propagation distance are 174 m and 36 m, respectively, when using the new large sample stage.
