# Supplementary material for: Evolution of hierarchical phase-contrast tomography on the European Synchrotron beamlines BM05 and BM18: a whole adult human brain imaging case study
Source: J Synchrotron Radiat. 2026 Jun 19;33(Pt 4):1180–9. doi: 10.1107/S1600577526005503 (PMC13344596; doi:10.1107/S1600577526005503)
Supplement: Supplementary file 2 [file s-33-01180-sup2.pdf]

JOURNAL OF SYNCHROTRON RADIATION

Volume 33 (2026)

Supporting information for article:

Evolution of Hierarchical Phase-Contrast Tomography on the European Synchrotron beamlines BM05 and BM18: a whole adult human brain imaging case study

Paul Tafforeau, Hector Dejea, Joseph Brunet, Theresa Urban, Camille Berruyer, Alessandro Mirone, Christoph Jarnias, Alexandre Bellier, Claire Walsh, Peter D. Lee and Paul Tafforeau

Table S1 Summary of all HiP-CT datasets acquired for the human brain across BM05 and BM18 beamlines. The table includes acquisition parameters (voxel size, propagation distance, exposure), scanning configurations, and corresponding dataset links (DOIs) for each experiment.

See gy5090sup1.xlsx for full table.

Table S2 Comparison of the key specifications and performance of the sCMOS detectors used in this study (PCO Edge 4.2 CLHS and Iris 15), including pixel size, sensor dimensions, field of view, dynamic range, and acquisition characteristics.

	PCO edge 4.2 CLHS	Teledyne Photometrics Iris 15
**Sensor type**	sCMOS	sCMOS (Gpixel GSense 5130)
**number of pixels**	2048 × 2048 (≈ 4.2 MP)	5056 × 2960 (≈ 15 MP)
**Pixel size**	6.5 µm	4.25 µm
**Sensor size**	≈ 13.3 × 13.3 mm (diag. ~18.8 mm)	≈ 21.5 × 12.6 mm (diag. ~24.9 mm)
**Max frame rate (full frame)**	100 fps	30 fps
**Readout noise**	~0.8 e^-	~1.5 e^-
**quantum efficiency at LuAG:CE emission peak (520nm)**	~82 %	~73 %
**Dynamic range**	~91 dB	~78 dB
**Full well capacity**	~30,000 e^-	~13,000 e^-
**Shutter**	Rolling (global reset)	Rolling + effective global shutter
**Interface**	CLHS	PCIe
**ADC resolution**	16-bit	16-bit
**Dark current**	<0.6 e^-/pix/s @7°C	~0.5 e^-/pix/s @0°C
**Cooling**	Air	Air

Table S3 Quantitative estimation of effective spatial resolution and image quality across HiP-CT configurations. Resolution metrics were derived using a patch-based Fourier Shell Correlation (FSC) analysis applied to reconstructed volumes (see Supplementary Methods). Reported values include the mean local FSC resolution, the best 5% local resolution (representing the most resolved regions), and global resolution estimates obtained from the average FSC curve using thresholds of 0.143 (standard criterion) and 0.05 (empirical criterion corresponding to the main signal-to-noise transition). Contrast-to-noise ratio (CNR) is provided as an additional metric of image quality. Values are reported as mean ± standard deviation across all valid patches.

Pixel size (µm)	Mean local FSC resolution (µm)	Best 5% local resolution (µm)	FSC resolution (0.143) (µm)	FSC resolution (0.05) (µm)	Contrast-to-noise ratio
**14.79**	29.0 ± 0.2	21.9 ± 0.3	32.5 ± 0.2	27.7 ± 0.3	11.0 ± 0.6
**19.28**	40.3 ± 0.9	34.0 ± 1.1	44.4 ± 0.9	38.7 ± 0.6	11.1 ± 1.2
**20.18**	40.4 ± 0.3	29.8 ± 0.2	45.2 ± 0.4	39.2 ± 0.6	14.2 ± 1.5
**23.42**	51.4 ± 1.2	43.8 ± 1.0	56.7 ± 0.8	48.9 ± 0.6	5.7 ± 0.2
**25.25**	62.6 ± 1.3	51.3 ± 0.8	68.7 ± 1.2	55.5 ± 0.6	7.8 ± 0.7
**42.40**	93.0 ± 2.3	64.1 ± 2.3	102.8 ± 1.2	86.0 ± 1.5	8.0 ± 0.7

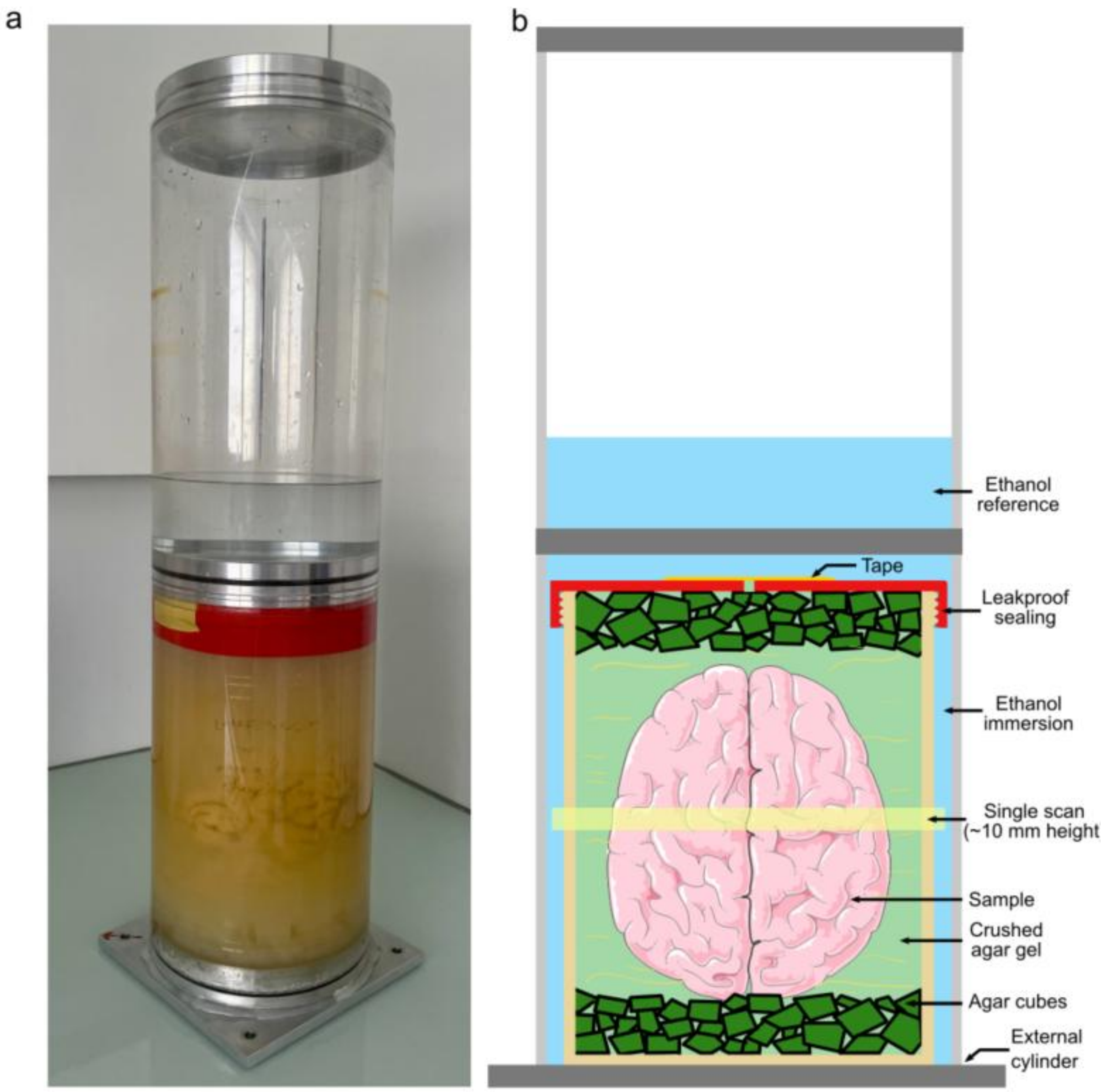

Figure S1 a) Picture and b) schematic of the organ mounting required for HiP-CT.

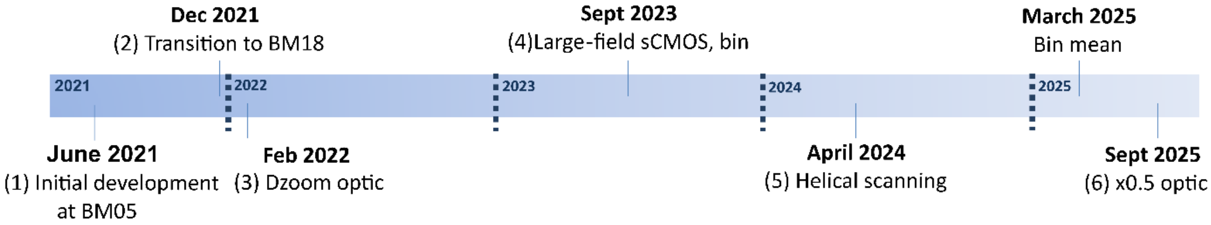

Figure S2 Timeline of development in whole adult human organ imaging at BM05 and BM18.

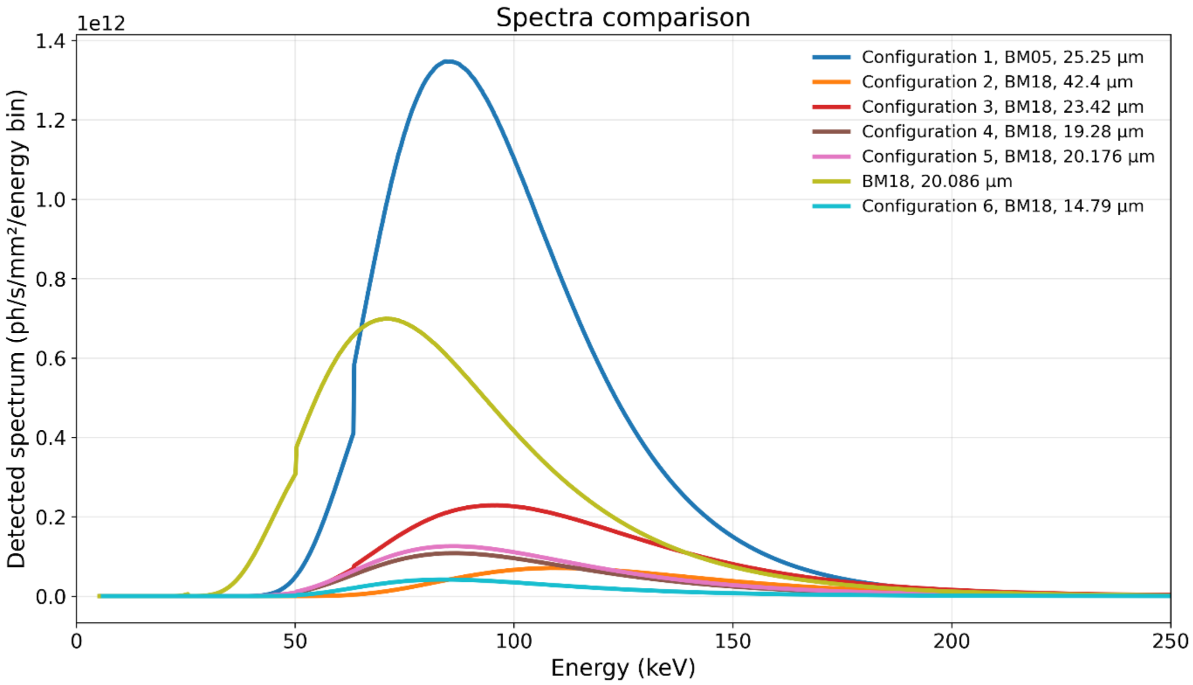

Figure S3 Detected X-ray spectra for all configurations, corresponding to the BM05 and BM18 acquisitions used in this study, and the bubbling configuration described in the Supplementary Excel Table S1. The figure illustrates the effect of the different filter combinations and beamline configurations on the detected energy distribution for each setup.

S1. Dose estimation

The polychromatic spectra of both beamlines are simulated in detail, including all beamline elements, air propagation, and sample properties. From this simulation, a theoretical water-equivalent surface dose rate is calculated for a given set of experimental conditions.

This calculation is performed according to the following protocol:

- Calculation of the photon flux at the sample surface, expressed in photons$\cdot$s$^{-1}\cdot$mm^{-2} per energy bin (energy bin = 100 eV, ranging from 100 eV to 800 keV).

- Calculation of the corresponding incident energy rate per mm² (J$\cdot$s^{-1} per energy bin). For each bin, this value is obtained by multiplying the photon energy (in eV) by the surface photon flux and by the conversion factor from eV to Joules (1.60218×10^{-19} J).

- The transmitted energy rate (J$\cdot$s^{-1}) through 100 µm of water is calculated for each energy bin using the mass energy-absorption coefficient (µ_en) of water.

- The water kerma is calculated for each energy bin by subtracting the transmitted energy rate from the incident energy rate, and then dividing by the reference mass of water crossed by the beam ($1 \times 1 \times 0.1$ mm^3, i.e. 1×10^{-7} kg).
- The water-equivalent surface dose rate (Gy·s^{-1}) is finally obtained by integrating the water kerma over the full spectrum.

These calculations provide a reasonably good estimate of the surface dose rate on the sample. To improve accuracy, dose rates were experimentally measured for 70 configurations on BM05 and BM18 using a TM31010 Semiflex ionization chamber coupled to a PTW Unidos E (T10021) dosimeter. These measurements cover the full range of energies and intensities available on both beamlines for microtomographic experiments. They were used to calibrate the simulation, yielding good agreement ($R^2 = 0.9973$ for dose rates > 1.5 Gy·s^{-1} and $R^2 = 0.9655$ below this threshold).

The integrated surface dose was calculated as the product of dose rate and total exposure time, accounting for scan geometry (half or quarter acquisition, vertical overlap, accumulation). In the case of sample immersion, the dose rate at the sample surface is reduced due to absorption by the external container and immersion liquid, which are treated as additional filters. This integrated surface dose is used to compare complete configurations, as the total dose depends on the spectrum, dose rate, and total exposure time.

The absorbed dose within the sample is estimated by calculating, for each energy bin, the transmitted energy rate through the entire sample, subtracting it from the incident energy rate, and dividing by the same reference water mass (1×10^{-7} kg). This value is then divided by the average sample density (g·cm^{-3}) in the case of ethanol-based preparation.

These calculations provide consistent estimates of dose for comparison between configurations, rather than precise dosimetry. For accurate absolute measurements, a calibrated dosimeter must be used during beamtime.

S2. Near-field limit and geometrical blur

As described in (Salditt *et al.*, 2017), the near-field regime for a sample structure of size a measured at a wavelength λ is given by a Fresnel number (F) below 1, so that as $F = \frac{a^2}{\lambda} < 1$. We assumed that at least 2 pixels are required to resolve a structure, so that the critical propagation distance z_c for a specific structure size is given by $z_c = \frac{(2*pixel\_size)^2}{\lambda}$.

Geometrical blur (g) is caused by the finite source size (σ) and the ratio of source-sample distance (*SSD*) and sample-detector distance (*SDD*). For this calculation, we assumed a maximum blur of 1.5 pixels, so that $g = \frac{\sigma}{1.5}\frac{SDD}{SSD}$.

Supplementary Figure S4 presents the near-field limit and geometrical blur for BM05 and BM18.

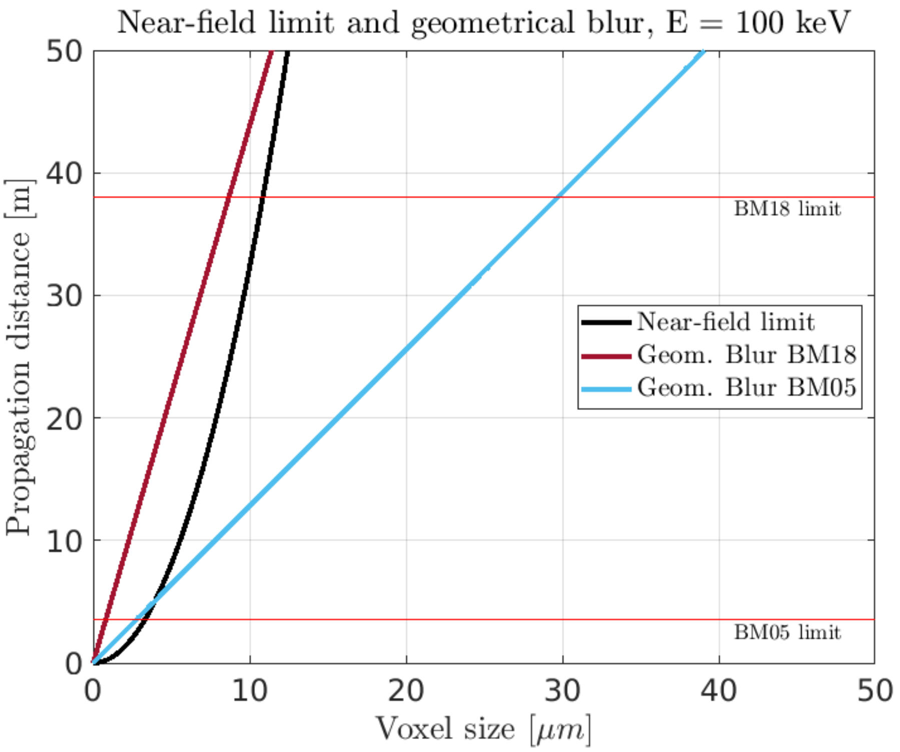

Figure S4 Near-field limit at an energy of 100 keV and geometrical blur curves for BM18 and BM05. Geometrical blur is independent of the energy. The horizontal red lines indicate the maximum propagation distance allowed by the experimental hutches.

S3. Resolution and Image Quality Estimation

S3.1. Estimation of effective spatial resolution

The effective spatial resolution of the reconstructed volumes was estimated using a patch-based Fourier correlation approach applied to 2D slices. This method provides local estimates of resolution and enables comparison across acquisitions with different voxel sizes.

For each dataset, a centered sub-volume corresponding to a fixed physical height (~5 mm) was extracted to ensure comparability between scans. Each slice was then subdivided into overlapping square patches defined in physical units (~5 mm), ensuring that all datasets were analysed over equivalent spatial regions. For each patch, a Fourier Ring Correlation (FRC)-based analysis was performed. Two sub-images were generated from a single image using an interleaved sampling

strategy (even/odd pixel splitting), and their Fourier transforms were computed. The normalized cross-correlation between the two sub-images was calculated:

$$\mathrm{FSC} = \frac{\Re(F_A \cdot F_B^*)}{\sqrt{|F_A|^2 \cdot |F_B|^2}}$$

A radial averaging was then performed to obtain a 1D FSC curve as a function of spatial frequency. The spatial resolution for each patch was determined from the FSC curve using a threshold-based criterion. The cut off frequency was defined as the first frequency at which the FSC drops below a threshold value. Two thresholds were used: (i) 0.143, corresponding to the standard criterion commonly used in Fourier correlation analysis, (ii) 0.05, used here as an empirical indicator of the transition between structured signal and noise in partially correlated data. This approach provides an estimate of effective resolution rather than an absolute system resolution and is primarily intended for relative comparison between configurations

S3.2. Contrast-to-noise estimation

A local contrast-to-noise ratio (CNR) was estimated for each patch to assess image quality independently of resolution. The signal component was estimated using a Gaussian low-pass filter (σ=0.5), while the noise component was defined as the high-frequency residual. The reported CNR values correspond to the ratio of the standard deviation of the signal to that of the noise.
